# An Indirect Comparison of Efficacy and Safety of Elvitegravir/Cobicistat/Emtricitabine/Tenofovir Disoproxil Fumarate and Abacavir/Lamivudine + Dolutegravir in Initial Therapy

**DOI:** 10.1371/journal.pone.0155406

**Published:** 2016-05-19

**Authors:** Josep M. Llibre, François Raffi, Graeme Moyle, Georg Behrens, Stephane Bouee, Geraldine Reilly, Peter Borg, David Piontkowsky, Felipe Rogatto

**Affiliations:** 1 University Hospital Germans Trias, Barcelona, Spain; 2 CMIT, Paris, France; 3 Chelsea and Westminster Hospital, London, United Kingdom; 4 Hannover Medical School, Hannover, Germany; 5 CEMKA-EVAL, Bourg-La-Reine, France; 6 Gilead Sciences, Stockley Park, United Kingdom; 7 Gilead Sciences, Foster City, United States of America; Rush University, UNITED STATES

## Abstract

**Objectives:**

The objective of this analysis is to perform an indirect comparison of elvitegravir, cobicistat, emtricitabine and tenofovir DF (E/C/F/TDF) to abacavir/lamivudine and dolutegravir (ABC/3TC + DTG) by using 2 trials evaluating each of these regimens in comparison to efavirenz, emtricitabine and tenofovir DF (EFV/FTC/TDF).

**Methods:**

An indirect comparison was performed by using a generalization of Bucher's methodology to calculate risk differences. Two phase III clinical trials (GS-US-236-0102 and SINGLE—described above) were used.

**Results:**

Results of the indirect comparison showed no statistically significant risk difference of the efficacy endpoint of achieving HIV RNA < 50 copies/mL between E/C/F/TDF and ABC/3TC + DTG for the ITT population at weeks 48, 96 and 144: respectively -3.7% (CI95% = [-10.8%; 3.4%]), -5.2% (CI95% = [-13.2%; 2.8%]) and -3.1% (CI_95%_ = [-12.0%; 5.7%]). There was no statistically significant differences in the risk difference for serious adverse events (5.7% (CI95% = [-2.2%; 12.3%])), drug related adverse event (2.7% (CI95% = [-7.0%;12.4%])), drug related serious adverse event (0.8% (CI95% = [-1.6%;3.2%])) and death (0.5% (CI95% = [-0.8%;1.8%])), respectively, between E/C/F/TDF and ABC/3TC + DTG. A significant difference was found for discontinuation due to adverse events with a higher rate for E/C/F/TDF (difference = 8.6% (CI95% = [3.3%; 13.9%])). There was also no statistically significant risk difference of the viral resistance of 1.2% (CI95% = [-1.2; 3.7]) between E/C/F/TDF and ABC/3TC + DTG at week 48, 1.7% at week 96 (CI95% = [-1.1; 4.5]) and 2.2% (CI95% = [-1.0; 5.4]) at week 144.

## Introduction

According to the Guidelines for the Use of Antiretroviral Agents in HIV-1-Infected Adults and Adolescents of the Department of Health and Human Services the initial antiretroviral therapy (ART) should combine two nucleos(t)ide reverse-transcriptase inhibitors (NRTIs) and another compound, which can be an integrase strand transfer inhibitor (dolutegravir, cobicistat boosted elvitegravir, or raltegravir), or the ritonavir-boosted protease inhibitor darunavir [1]. Two integrase inhibitors are now available as single tablet regimens (STR) with two NRTIs and the integrase inhibitor [2,3]. These drugs were recently made available for HIV treatment and have good efficacy and safety results [4,5].

The co-formulated regimens of elvitegravir/cobicistat/emtricitabine/tenofovir disoproxil fumarate (E/C/F/TDF) and abacavir/lamivudine + dolutegravir (ABC/3TC + DTG) have been extensively studied in initial ART therapy. On a total of 1,067 participants receiving ABC/3TC + DTG, 88.3% (942) had an HIV-1 RNA < 50 copies/mL at 48 weeks [6,7,8]. On a total of 1,422 participants receiving E/C/F/TDF, 90.7% (1,567) had an HIV-1 RNA < 50 copies/mL at 48 weeks [9,10,11]. But E/C/F/TDF and ABC/3TC + DTG have never been compared head to head in a randomized trial. However these compounds have been compared to EFV/FTC/TDF in two double-blind randomized trials in treatment-naïve patients:

- SINGLE included 833 patients in 2010 [6,12];
- GS-US-236-0102 included 700 patients in 2011 [9,13].

Despite showing similar rates of efficacy among the integrase inhibitors, the different rates of efficacy of EFV/FTC/TDF in both studies (higher rates of discontinuation due to adverse events [10 vs 5%] in the SINGLE vs GS-US-236-0102) established superiority of ABC/3TC + DTG and non-inferiority of E/C/F/TDF against EFV/FTC/TDF. Both studies have now reported data at 48, 96 and 144 weeks.

The randomized controlled trial is the gold standard for evaluating the relative efficacy of healthcare technology. However, many competing interventions have not been directly compared in randomized clinical trial and indirect treatment comparisons have been advocated when direct evidence is not available [14]. An indirect comparison refers to a comparison of different healthcare interventions using data from separate studies (in contrast to a direct comparison within randomised controlled trials). Indirect comparison is often performed in case of insufficient evidence from head-to-head comparative trials. These types of comparisons are becoming more commonplace, partly because Health Authorities are requisting this kind of studies. In France for example, specific guidelines to perform indirect comparisons were developed by the *Haute Autorité de Santé* (HAS) in 2009 [15].

The objective of this analysis was to perform an indirect treatment comparison of the efficacy, safety, rates of virological failure and emergence of resistance at 48, 96 and 144 weeks of E/C/F/TDF to ABC/3TC + DTG by using these two randomized trials which compared each of these regimens to EFV/FTC/TDF.

The studies compared here were conducted at one year interval (2010 and 2011), and recruited participants with similar baseline characteristics.

## Methods and Data

### The analysis was performed on aggregated data: patient records/information was anonymized and de-identified prior to analysis

None of the authors had access to identifying patient information. Therefore, there was no need for an ethic statement

For ABC/3TC + DTG (study SINGLE) the aggregated data were extracted from:

- The publications of Walmsley et all [16] for the results at week 48;
- The communication and the publication of Walmsley et al [16,17] for results at week 96;
- The publication of Walmsley et al and the communication of Pappa et al. [18, 19] for results at week 144.

For E/C/F/TDF (study GS-US-236-0102) the aggregated data were extracted from:

- The publications of Sax et all [14] for the results at week 48;
- The publications of Zolopa et all [19] for the results at week 96;
- The communication and the publication of Wohl et al. [20,21] and the communication of Cohen et al [22] for results at week 144.

**Fig 1:**
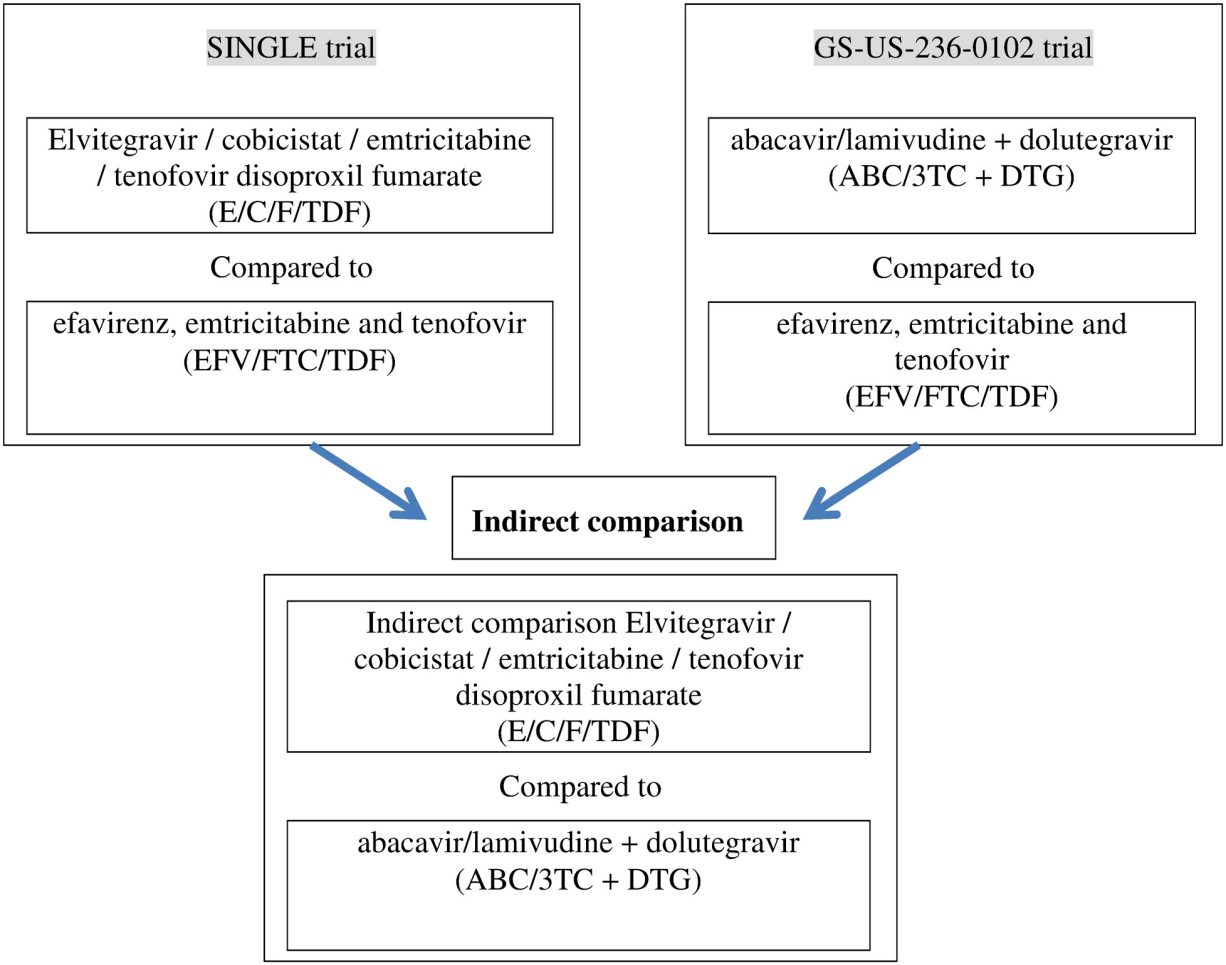
Flowchart of the indirect comparison analysis.

These studies had similar designs and compared E/C/F/TDF or ABC/3TC + DTG versus EFV/FTC/TDF (Fig 1). Both were phase III, double-blind, multicenter, active-controlled randomized trials, having as their primary endpoint the proportion of individuals with HIV-1 RNA < 50 copies/mL at week 48 (“virologic success”) based on an ITT FDA snapshot analysis. Both included treatment-naïve subjects with similar baseline characteristics (Table 1), although GS-US-236-0102 enrolled more Blacks and Hispanics.

**Table 1:** Baseline characteristics of patients included in the 2 studies.

	E/C/F/TDF	ABC/3TC + DTG		EFV/FTC/TDF		
	GS-US-236-0102	SINGLE Study		GS-US-236-0102	SINGLE Study	
	(N = 348)	(n = 414)	p	(N = 352)	(n = 419)	p
Age in years (Median)	37.0	36.0	*	38.0	35.0	*
Gender			0.08			0.04
Male	307 (88%)	347 (84%)		316 (90%)	356 (85%)	
Female	41 (12%)	67 (16%)		36 (10%)	63 (15%)	
Race			<0.001			<0.001
• White	132 (37%)	228 (55%)		142 (40%)	229 (55%)	
• Hispanic	82 (24%)	56 (14%)		85 (24%)	56 (13%)	
• Black	106 (30%)	98 (24%)		91 (26%)	99 (24%)	
• Other	28 (9%)	32 (7%)		34 (9%)	32 (7%)	
HCV positive	17 (5%)	27 (7%)	0.3	15 (4%)	29 (7%)	0.1
Plasma HIV-1 RNA (copies per mL)			0.6			0.6
• ≤100 000	230 (66%)	280 (68%)		236 (67%)	288 (69%)	
• >100 000	118 (34%)	134 (32%)		116 (33%)	131 (31%)	
CD4 Cell Count (/μL)						
Median	376	335	*	383	339	*
• ≤ 50	7 (2%)	13 (3%)		6 (2%)	14 (3%)	
• 51 to ≤200	36 (10%)	44 (11%)	0.6	45 (13%)	48 (11%)	0.3
• >200	305 (87%)	357 (86%)		301 (86%)	357 (85%)	

* Cannot be calculated.

In the SINGLE study, randomization was stratified according to the plasma HIV-1 RNA level at the time of screening (≤100,000 copies per milliliter vs. >100,000 copies per milliliter) and the CD4+ T-cell count (≤200 per cubic millimeter vs. >200 per cubic millimeter). In the study GS-US-236-0102, randomization was only stratified by HIV RNA concentration at screening (≤100,000 copies per milliliter vs. >100,000 copies per milliliter). In both studies eligible patients were randomised in a 1:1 ratio.

The GS-US-236-0102 remained double blinded until week 144 but the SINGLE study was unblinded at week 96 and continued through week 144.

Participants in both studies were adults infected with HIV-1, aged at least 18 years with plasma HIV-1 RNA concentrations of 5,000 copies/mL or more (GS-US-236-0102) or 1,000 copies/mL or more (SINGLE) and no previous use of antiretroviral drugs. There was no screening CD4 cell count limit in the inclusion criteria. Participants had to have an estimated glomerular filtration rate of at least 70 mL/min (GS-US-236-0102) or 50 mL/min (SINGLE) and be susceptible to all study drugs by HIV-1 genotype at screening. Participants in the SINGLE study were negative for the HLA-B*5701 allele. Randomization was stratified according to the plasma HIV-1 RNA level at the time of screening (≤100,000 copies per milliliter vs. >100,000 copies per milliliter) in both studies, and also to the CD4+ T-cell count (≤200 per cubic millimeter vs. >200 per cubic millimeter) in the SINGLE study.

In the snapshot analysis, participants with HIV-1 RNA less than 50 copies/mL between days 309 and 378 (week 48 window) were classified as virological success in the GS-US-236-0102 study, and between week 42 through week 54 in SINGLE.

Participants with missing HIV-1 RNA data for weeks 48, 96 and 144 analysis window, who discontinued study drug, or who changed therapy being suppressed at the previous time points were considered as having no data in the window.

Virological failure was defined as HIV RNA > 50 copies/mL after week 8 in the GS-US-236-0102 study and after week 24 in SINGLE. For those patients failing, genotype testing was conducted in the confirmatory sample (second sample) if HIV-1 RNA > 400 copies/mL in GS-US-236-0102. In the SINGLE study, genotyping test was conducted in the first sample showing HIV RNA > 50 copies/mL. We decided to analyze the resistance emerging at virological failure in both studies at 144 weeks to allow the maximal number of failures and resistance tests done [23].

Finally, 707 patients were enrolled in the GS-US-236-0102 study and 700 (99%) contributed to the ITT population, and 844 patients were enrolled in the SINGLE study, with 833 (99%) receiving study drug and contributing to the ITT population.

An indirect method proposed by Bucher et al. in 1997 has been broadly utilized for indirect treatment comparisons in meta-analyses of RCTs for discrete data [24].

This model was developed with the odds ratio (OR) as the measure of treatment effect. However, OR are used for ratio calculations and we could not use this method for the calculations of differences. Therefore, a generalization of the Bucher approach that allows indirect comparisons using other measures of association, including risk differences, has been recommended [25].

Comparisons between treatments were made using a risk difference measure:

1. A risk difference (RD_1_) has been calculated between E/C/F/TDF and EFV/FTC/TDF: RD = P_E/C/F/TDF_ − P_EFV/FTC/TDF_, where P_E/C/F/TDF_ is the probability of virologic success, development of viral resistance or safety parameters for E/C/F/TDF and P_EFV/FTC/TDF_ for EFV/FTC/TDF;
2. A risk difference (RD_2_) has been calculated between ABC/3TC + DTG and EFV/FTC/TDF: RD_2_ = P_ABC/3TC + DTG_ − P_EFV/FTC/TDF_, where P_ABC/3TC + DTG_ is the probability of virologic success, development of viral resistance or safety parameters for ABC/3TC + DTG and P_EFV/FTC/TDF_ for EFV/FTC/TDF;
3. The 2 preceding result RD_1_ and RD_2_ are combined by subtracting the first by the second: RD = RD_1_ − RD_2_ (it is a difference of difference). The result represents the indirect difference between E/C/F/TDF and ABC/3TC + DTG.

When the risk difference percentage is greater than 0, this means that the proportion is higher for E/C/F/TDF as compared to ABC/3TC + DTG:

- For efficacy, a positive result means that a higher proportion of patients on E/C/F/TDF reached the endpoint of HIV-1 RNA < 50 copies/mL when compared to ABC/3TC + DTG;
- For resistance, a positive result means that a higher proportion of patients on E/C/F/TDF developed resistance when compared to ABC/3TC + DTG;
- For safety, a positive result means that a higher proportion of patients on E/C/F/TDF experienced adverse events when compared to ABC/3TC + DTG,

If the risk difference percentage is lower than 0, this means that the proportion is higher for ABC/3TC + DTG in comparison to E/C/F/TDF.

## Results

Most of the baseline characteristics of the study populations in the integrase arms of both clinical trials were similar (Table 1). There were significant differences in race, with a lower proportion of white patients in the GS-US-236-0102 trial compared to the SINGLE study: 37% versus 55% (p<0.001). There were no significant differences in other baseline characteristics potentially associated with virological response: gender, HIV-1 plasma RNA, CD4 cell count or co-infection with viral hepatitis.

### Efficacy

**Fig 2:**
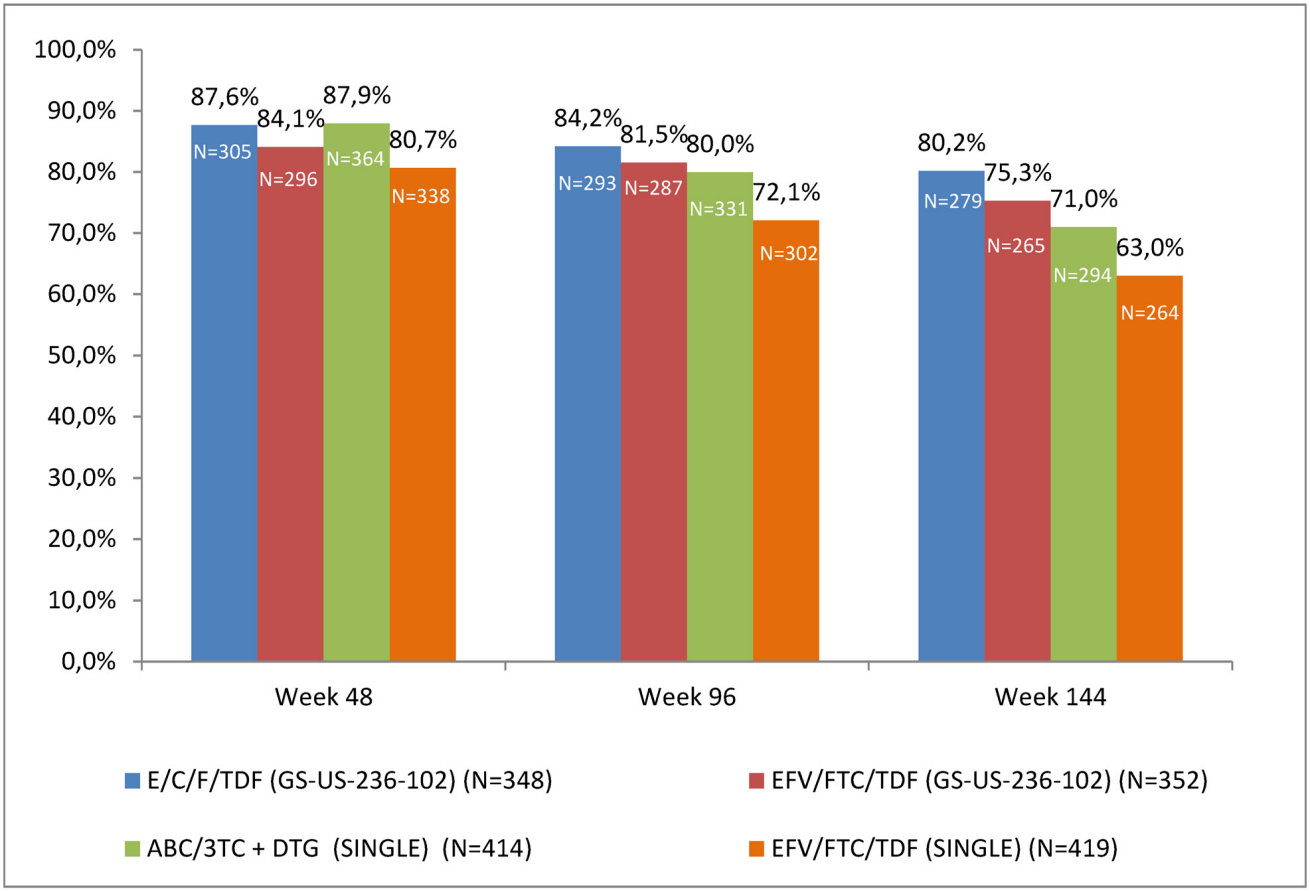
Proportion of patients with HIV-1 RNA < 50 copies/mL (ITT FDA snapshot analysis) at weeks 48, 96 and 144.

Overall results for efficacy are shown in Fig 2. The proportion of patients with plasma HIV-1 RNA < 50 copies/mL at weeks 48, 96 and 144 were higher for both investigational integrase regimens (E/C/F/TDF and ABC/3TC + DTG) as compared to EFV/FTC/TDF. The proportions of subjects with plasma HIV-1 RNA < 50 copies/mL was 87.6% with E/C/F/TDF and 87.9% with ABC/3TC + DTG at week 48, 84.2% and 80.0% at week 96 and 80.2% and 71.0% respectively at week 144 (Fig 2).

**Fig 3:**
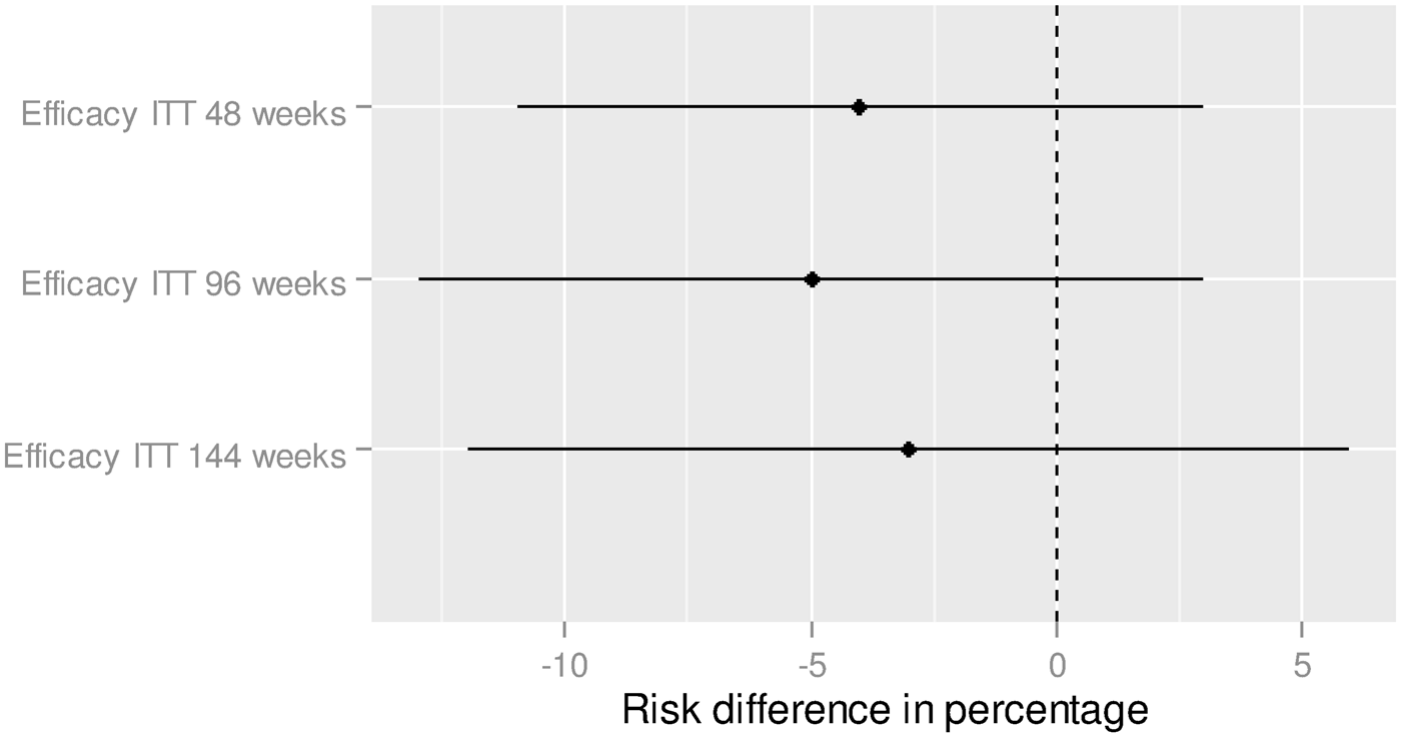
Efficacy,—Indirect treatment comparison—E/C/F/TDF vs. ABC/3TC + DTG. To the right of zero favors ABC/3TC/DTG (If the risk difference percentage is higher (lower) than 0, this means that the proportion is higher (lower) for ABC/3TC + DTG in comparison to E/C/F/TDF).

Results of the indirect comparison showed no statistically significant difference between E/C/F/TDF and ABC/3TC + DTG in the adjusted risk difference (relatively to the control group) of the efficacy endpoint of achieving HIV RNA < 50 copies/mL at 48 weeks: -3.7% (CI95% = [-10.8%; 3.4%]) (Fig 3). The adjusted difference was -5.2% (CI95% = [-13.2%;2.8%]) at 96 weeks, and -3.1% (CI95% = [-12.0%; 5.7%]) at week 144.

### Safety

**Fig 4:**
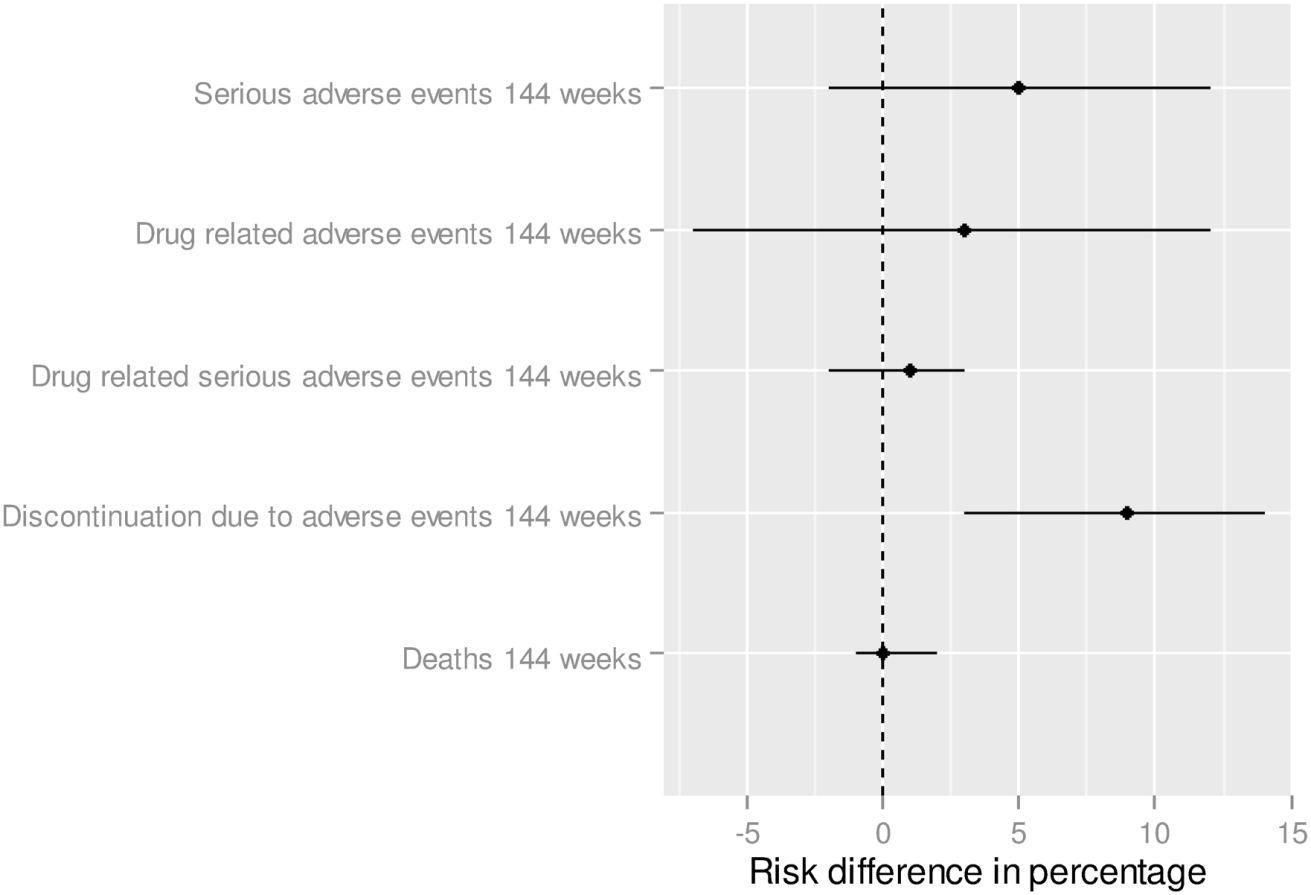
Safety—Indirect treatment comparison—E/C/F/TDF vs. ABC/3TC + DTG. To the right of zero favors E/C/F/TDF (If the risk difference percentage is higher (lower) than 0, this means that the proportion is higher (lower) for ABC/3TC + DTG in comparison to E/C/F/TDF).

The analysis did not show statistically significant differences between E/C/F/TDF and ABC/3TC + DTG in the risk difference for serious adverse events: +5.7% (CI95% = [-2.2%; 12.3%]) (Fig 4). The adjusted difference was 2.7% (CI95% = [-7.0%;12.4%]) for drug related adverse event, 0.8% (CI95% = [-1.6%;3.2%]) for drug related serious adverse event, 0.5% (CI95% = [-0.8%;1.8%]) for death and 8.6% (CI95% = [3.3%; 13.9%]) for discontinuation due to adverse events.

### Virological failure and resistance

Rates of virological failure were comparable in both studies. In GS-US-236-0102, 7% of patients on E/C/F/TDF and 10% of subjects on EFV/FTC/TDF were considered virological failures at week 144, while in SINGLE, rates were 10% and 7% for ABC/3TC + DTG and EFV/FTC/TDF, respectively.

As pointed out previously, resistance was analysed differently in GS-US-236-0102 and SINGLE. While 2.9% (10/348) patients on E/C/F/TDF in GS-US-236-0102 and no patients on ABC/3TC + DTG in SINGLE developed resistance up to week 144, the proportion of patients on EFV/FTC/TDF developing resistance at week 144 were 3.9% and 1.4% in GS-US-236-0102 and SINGLE, respectively.

**Fig 5:**
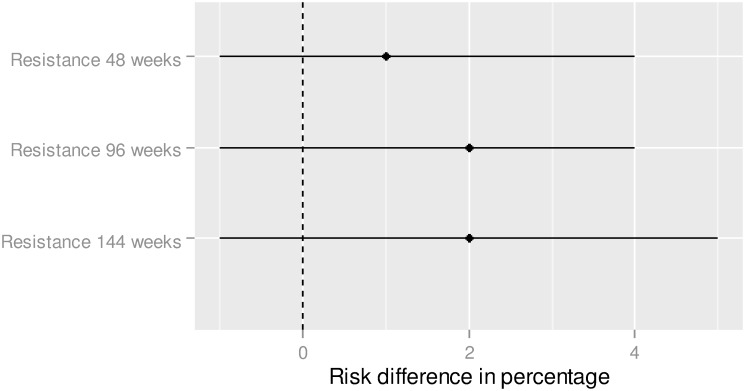
Resistance—Indirect treatment comparison—E/C/F/TDF vs. ABC/3TC + DTG. To the right of zero favors E/C/F/TDF (If the risk difference percentage is higher (lower) than 0, this means that the proportion is higher (lower) for ABC/3TC + DTG in comparison to E/C/F/TDF).

Results of the indirect comparison showed no statistically significant risk difference of the viral resistance of 1.2% (CI95% = [-1.2; 3.7]) at week 48, 1.7% at week 96 (CI95% = [-1.1; 4.5]) and 2.2% (CI95% = [-1.0; 5.4]) at week 144 (Fig 5).

## Discussion

Indirect comparison analysis are recommended by the French, Canadian and UK guidelines in case of no direct clinical studies, but are not very commonly applied in regulatory submissions [26,27]. However, the number of available anti-HIV treatments is larger than the number of recommended treatments, as newer options with greater convenience, tolerability and potency, have replaced previous treatments as first-line options. In the last DHHS Panel on Antiretroviral Guidelines, treatment with EFV/FTC/TDF has been moved to the alternative regimens category because of concerns about the tolerability of EFV in clinical trials and practice, especially the high rate of central nervous system (CNS)-related toxicities and a possible association with suicidality [28].

Our indirect comparison was aimed to compare the efficacy, emergence of resistance and safety of E/C/F/TDF to ABC/3TC + DTG. The results of two randomized trials using the same comparator (EFV/FTC/TDF) were analysed. The results show equivalence between E/C/F/TDF and ABC/3TC + DTG in terms of efficacy, resistance and most safety results.

These results are in accordance with previous works. In a network meta-analysis, Patel et al compared several nucleoside reverse transcriptase inhibitor regimens [29]. The authors evaluated week 48 efficacy (HIV-RNA suppression to <50 copies/mL and change in CD4+ cells/μL) and safety (discontinuations due to adverse events, adverse events and laboratory abnormalities) of DTG relative to all other treatments. The results did not show a statistical differences between E/C/F/TDF and ABC/3TC + DTG in terms of efficacy at 48 weeks. In terms of safety, AEs were not significantly different between E/C/F/TDF and ABC/3TC + DTG, but discontinuation due to AEs was significantly higher with E/C/F/TDF compared to ABC/3TC + DTG [30]. These results are in accordance with our findings at 144 weeks.

Although the 2 studies we analyzed were very similar in their methodologies, the GS-US-236-0102 remained blinded until week 144 but the SINGLE study was unblinded at week 96. This difference may have biased some of the analysis we have performed. In GS-US-236-0102 the proportion of patients reported as having ‘No data’ for E/C/F/TDF and EFV/FTC/TDF were 5% and 9% at week 48, 9% and 11% at week 96, and 12% and 15% at week 144. In the SINGLE trials these percentages were 7% and 13% at week 48, 12% and 20% at week 96, and 18% and 30% at week 144 respectively for ABC/3TC + DTG and EFV/FTC/TDF.

The randomizations were quite similar and we believe that the differences did not bias the indirect comparison analysis. In the study GS-US-236-0102, participants had to have an estimated glomerular filtration rate of at least 70 mL/min and in the SINGLE study, persons with an estimated creatinine clearance of less than 50 ml per minute were excluded from the study. Hereto, we believe that these differences did not generate a significant bias the indirect comparison analysis.

Most of the baseline characteristics of the study populations in the integrase arms of both clinical trials were similar. However, there were significant differences in race, with a lower proportion of white patients in the GS-US-236-0102 trial compared to the SINGLE study. The relation between race and efficacy of AIDS drugs has been questioned. However, the causes of this relation seem rather due to cultural and social factors which are correlated with race, rather than race itself [31]. Therefore, we believe that the small differences in the baseline characteristics would not be likely to cause an important impact in the results of this indirect comparison. However, we cannot rule out that these differences could have a possible impact on the results of our analysis.

There was a high dropout rate in the EFV/FTC/TDF arm of the SINGLE trial between week 96 and 144 (10% (30%-20%)) as compared to the study GS-US-236-0102 (4% (15%-11%)). This higher dropout rate may be due to the open label of the SINGLE trial at week 96 (patients taking the old treatment may want to be switched to the experimental one). This may have increased the difference in virologic success between ABC/3TC+DTG and EFV/FTC/TDF (as compared to E/C/F/TDF). Hence, as our calculations were based on the risk differences between both studies, this may have induced a higher difference in virologic success, although not statistically significant.

This difference between the two clinical trials may also explain some results observed for the indirect safety analysis. The higher proportion of patients experiencing a discontinuation of E/C/F/TDF (compared to ABC/3TC+DTG) at week 144 may also be explained by the high difference of dropout rate between week 96 and 144 for EFV/FTC/TDF in the SINGLE study.

In regards to discontinuations, the higher rate of dropouts for E/C/F/TDF in GS-US-236-0102 could initially be related to elevation in serum creatinine due to inhibition of the renal transporter MATE-1. This phenomenon was clearly understood later in time and was also found in the DTG studies, and investigators felt more comfortable continuing patients with these changes as they gained experience with the medications.

Many competing interventions have not been directly compared in randomized clinical trial and indirect methods have been commonly used in meta-analyses.

Several reasons can explain the lack of direct comparisons. Cost and time of conducting clinical studies accompanied with the fact that standard of care can change quickly are reasons to look for alternate solutions such as indirect comparisons to get an assessment of the true level of effect of the new drug [27]. Moreover, for regulatory approval, placebo-controlled trials are usually sufficient for acquiring of a new treatment. Therefore, head-to-head comparisons of active treatments are more rarely performed. In these situations, indirect treatment comparison can be of interest.

As for clinical trials, one should make his own point of view from what is statistically significant for the clinical interest of the results. Besides that, the absence of a statistical significant result does not imply an absence of the difference and absence of evidence is not evidence of absence. The U.S. Department of Health and Human Services has provided guidelines for non-inferiority and superiority clinical trials for HIV treatments [30]. These recommendations can be used to interpret the results in term of clinical signification.

There are limitations in the method that we used.

We only worked on statistical aggregated results of data because we did not have access to the individual data of both trials. We did not perform a systematic review. We identified these 2 studies as very similar and we believe that it would be interesting to perform this indirect comparative analysis. There are other clinical studies on integrase inhibitors (including Raltegravir), but this drug is not available as a STR or QD posology and the included populations are quite different across studies, which complicate the analysis. Finally we preferred to compare these 2 similar studies rather than including more studies which would have needed questionable adjustments.

The results we obtained depend on the quality and comparability of the populations included in the trials used to make the analysis. The studies we used are similar in terms of patients’ characteristics and the methodologies used to conduct the two studies were consistent. Only data corresponding to both, the definition for the treatment of missing data and of the virologic suppression were included in the analysis to ensure comparability of results. However, as stated above, the fact that study GS-US-236-0102 remained blinded until week 144 but the SINGLE study was unblinded at week 96 may have biased some indirect comparisons analysis.

## Conclusion

With the limitation that we did not perform a systematic review we can conclude that:

- The indirect efficacy comparisons show equivalence between E/C/F/TDF and ABC/3TC + DTG. For efficacy, the difference between both regimens at week 48, 96 and 144 were small and not statistically significant;
- Resistance and all safety results (except for discontinuation due adverse events) also show equivalence between the 2 regimens.

Indirect comparative data between active drugs, such as these results, assist clinicians in utilizing these agents in real life practice.

## Supporting Information

S1 Appendix Raw data and calculations. (XLS)
