# Correction: An Indirect Comparison of Efficacy and Safety of Elvitegravir/Cobicistat/Emtricitabine/Tenofovir Disoproxil Fumarate and Abacavir/Lamivudine + Dolutegravir in Initial Therapy

**DOI:** 10.1371/journal.pone.0159286

**Published:** 2016-07-08

**Authors:** Josep M. Llibre, François Raffi, Graeme Moyle, Georg Behrens, Stephane Bouee, Geraldine Reilly, Peter Borg, David Piontkowsky, Felipe Rogatto

The first author’s name appears incorrectly in the citation. The correct citation is: Llibre JM, Raffi F, Moyle G, Behrens G, Bouee S, Reilly G, et al. (2016) An Indirect Comparison of Efficacy and Safety of Elvitegravir/Cobicistat/Emtricitabine/Tenofovir Disoproxil Fumarate and Abacavir/Lamivudine + Dolutegravir in Initial Therapy. PLoS ONE 11(5): e0155406. doi:10.1371/journal.pone.0155406.

The Abstract is missing a sentence at the end of the “Results” subheading. The additional sentence is: In an indirect comparison, we found no statistically significant differences in efficacy, serious adverse events, drug related adverse events, drug related serious adverse events, death or selection of viral resistance between E/C/F/TDF and ABC/3TC + DTG in initial therapy.

There are numerical errors in the second sentence of the first paragraph in the Results section under the subheading “Virological failure and resistance.” The correct sentence is: In GS-US-236-0102, 7% of patients on E/C/F/TDF and 10% of subjects on EFV/FTC/TDF were considered virological failures at week 144, while in SINGLE, rates were 9% and 8% for ABC/3TC + DTG and EFV/FTC/TDF, respectively.

There are errors in the first two sentences of the Conclusion. The correct sentences are: With the limitation that we did not perform a systematic review we can conclude that: The indirect efficacy comparisons do not show significant differences between E/C/F/TDF and ABC/3TC + DTG. For efficacy, the difference between both regimens at week 48, 96 and 144 were small and not statistically significant; Resistance and all safety results (except for discontinuation due adverse events) also do not show significant differences between the 2 regimens.

The captions for Figs 3, 4 and 5 appear incorrectly in the published article. Please see Figs 3, 4 and 5, and their correct captions here.

**Fig 3:**
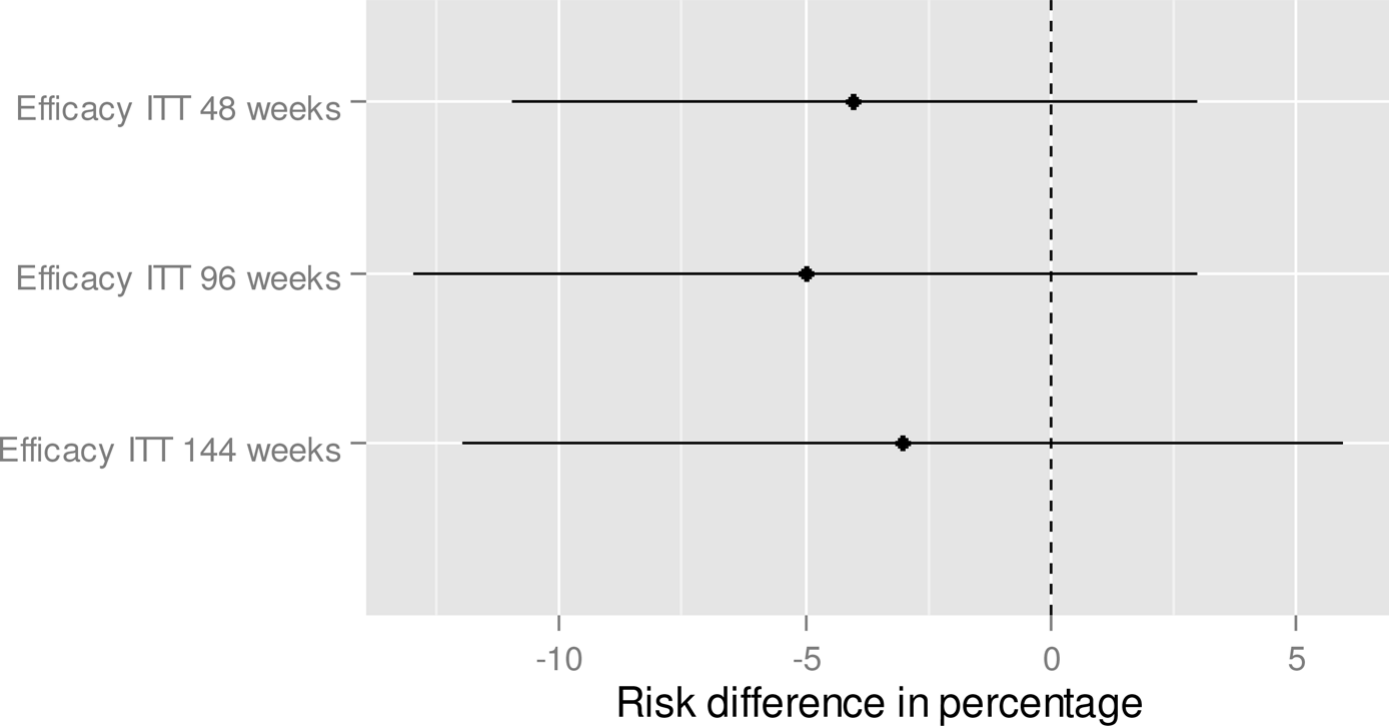
Efficacy—Indirect treatment comparison—E/C/F/TDF vs. ABC/3TC + DTG. To the right of zero favors E/C/F/TDF (if the risk difference percentage is higher than 0, this means that the proportion is higher for E/C/F/TDF in comparison to ABC/3TC + DTG).

**Fig 4:**
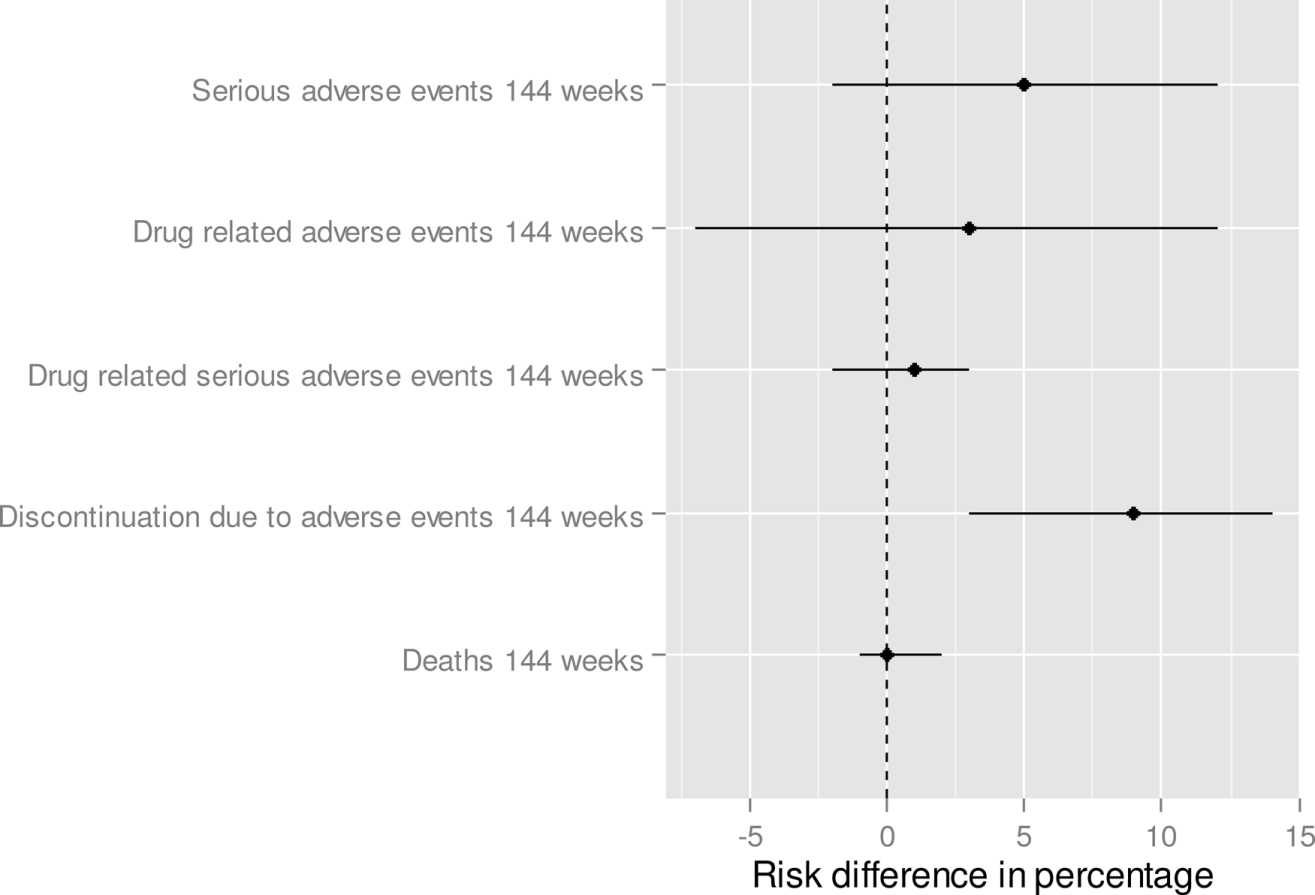
Safety—Indirect treatment comparison—E/C/F/TDF vs. ABC/3TC + DTG. To the right of zero favors ABC/3TC + DTG (if the risk difference percentage is higher than 0, this means that the proportion of toxicity is higher for E/C/F/TDF in comparison to ABC/3TC + DTG).

**Fig 5:**
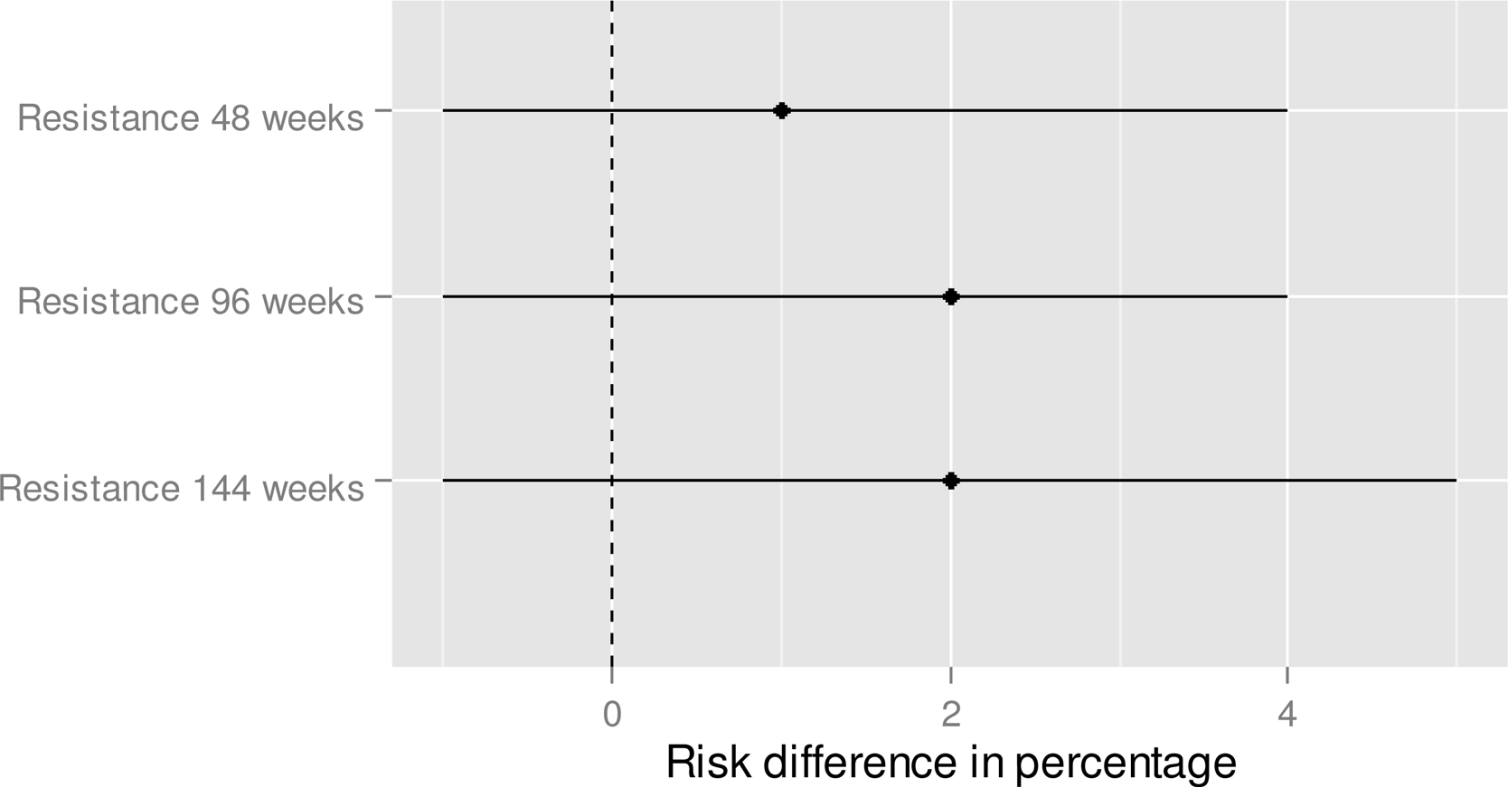
Resistance—Indirect treatment comparison—E/C/F/TDF vs. ABC/3TC + DTG. To the right of zero favors ABC/3TC + DTG (if the risk difference percentage is higher than 0, this means that the proportion of resistance is higher for E/C/F/TDF in comparison to ABC/3TC + DTG).
